# Poisoning by the White of an Egg

**Published:** 1904-07

**Authors:** 


### POISONING BY THE WHITE OF AN EGG.

That the idiosyncrasy of poisoning by white of egg must be a very rare one is evidenced by the fact that no mention is made of it in literature, says Dr. J. R. Clemens, of St. Louis, Mo. A patient of his, a child of fourteen months, was suddenly seized with an attack of acute urticaria and alarming collapse, the face and ears being greatly swollen and the radial pulse withdrawn from the wrist. The child was apparently moribund, but hypodermics of strychnine tided over the crisis. Later the child vomited the contents of the stomach—water and mucus. The pupils were widely dilated during the attack and the face pale. The child rallied slowly, and was the source of anxiety for the following two days.

A month later the child, after partaking of some custard, suffered within half an hour from an acute attack of urticaria of the face and ears, with vomiting and purging. The cause of the attack was now evident. Three days before, after eating a small piece of ginger-bread, in the making of which two eggs had been used, the child was noticed to be limping, and on examining the feet both were found to be extremely swollen and purpuric, petechiæ and ecchymoses extending up as high as the middle of the legs. There were no nervous symptoms.

Treatment consisted in cleaning out the gastro-intestinal tract with oil, followed by full doses of calomel as an antidote, and later by salol and magnesium sulphate. The recovery was uneventful, the edema of the feet rapidly disappearing, although the child was allowed to stand and walk. A short time prior to each of the first two alarming attacks the child had been given the white of an egg with his milk, which was an innovation.—*Medical News, April 16, 1904.*

